# Supplementary material for: Crystal-facet-dependent surface transformation dictates the oxygen evolution reaction activity in lanthanum nickelate
Source: Nat Commun. 2023 Dec 13;14:8284. doi: 10.1038/s41467-023-43901-z (PMC10719283; doi:10.1038/s41467-023-43901-z)
Supplement: Supplementary file 1 — Supplementary Information [file 41467_2023_43901_MOESM1_ESM.pdf]

Crystal-facet-dependent surface transformations dictate binding energetics and electrocatalytic activity in lanthanum nickel perovskite oxides Supplementary Information

Achim Füngerlings[1], Marcus Wohlgemuth[2], Denis Antipin[4], Emma van der Minne[3], Ellen Marijn Kiens[3], Javier Villalobos[4], Marcel Risch[4], Felix Gunkel[2], Rossitza Pentcheva[1], Christoph Baeumer[2,3]

November 16, 2023

[1]Department of Physics, Theoretical Physics and Center for Nanointegration (CENIDE), University of Duisburg-Essen, Duisburg, Germany
[2]Peter Gruenberg Institute and JARA-FIT, Forschungszentrum Juelich GmbH, Juelich, Germany
[3]MESA+ Institute for Nanotechnology, Faculty of Science and Technology, University of Twente, Enschede, Netherlands
[4]Nachwuchsgruppe Gestaltung des Sauerstoffentwicklungsmechanismus, Helmholtz-Zentrum Berlin für Materialien und Energie GmbH, 14109 Berlin, Germany;

1 Supplementary Note 1: Additional structural and compositional analysis of the as-prepared state.

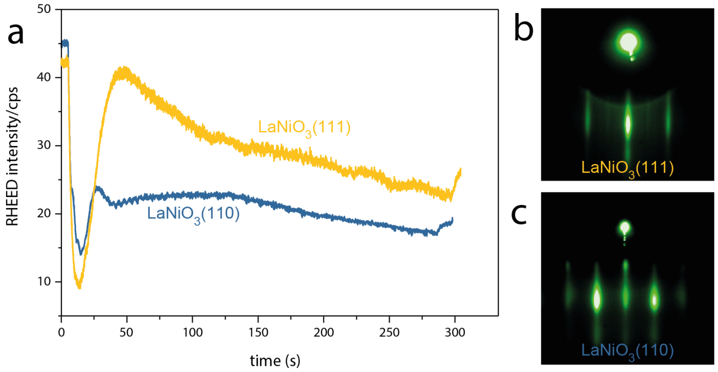

Supplementary Figure 1: **(a)** RHEED intensity evolution during PLD growth of $LaNiO_{3-\delta}$ on (110)- and (111)-oriented $SrTiO_3$ substrates. **(b, c)** RHEED patterns after growth, confirming two-dimensional growth.

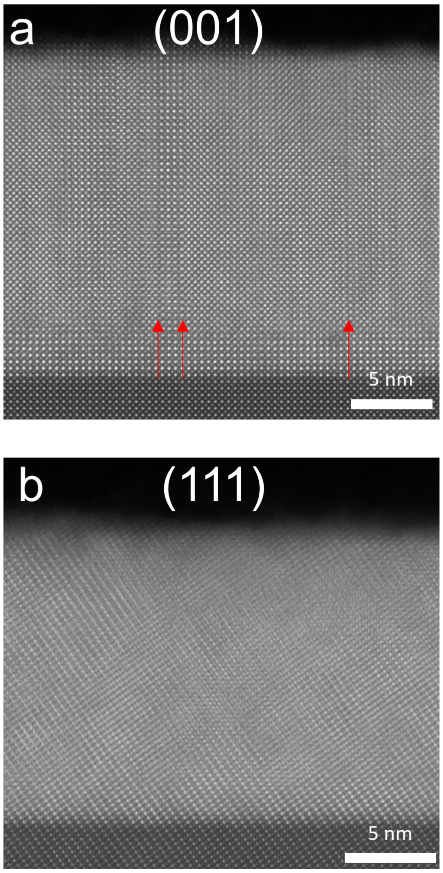

Supplementary Figure 2: HAADF STEM micrographs of $LaNiO_{3-\delta}$ films on **(a)** (001)- and **(b)**(111)-oriented $SrTiO_3$ substrates. We observe extended defects in the bulk of the film (examples highlighted with red arrows). These were observed before,[1] connected to the chosen synthesis temperature, which was in turn necessary to obtain the desired Ni-termination in the as-prepared state.

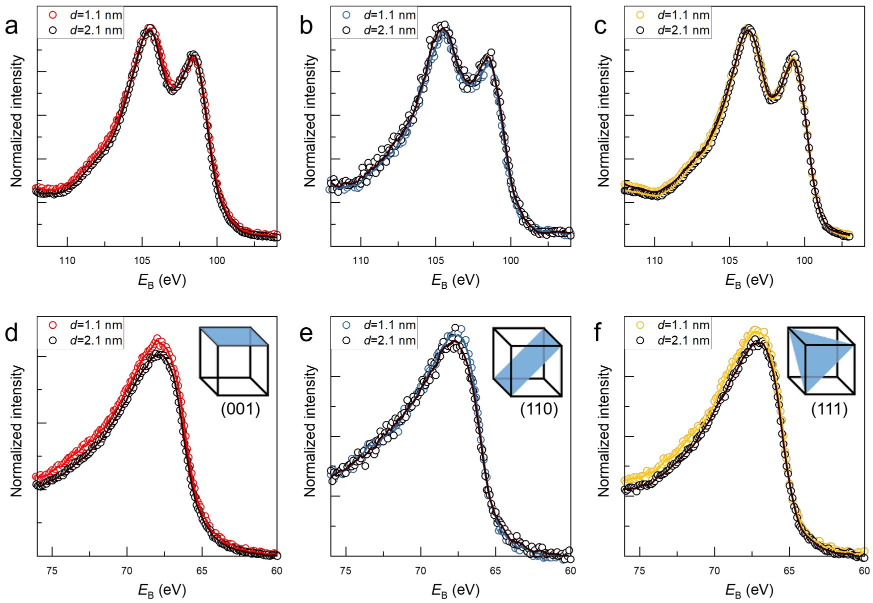

Supplementary Figure 3: **(a)-(c)** La 4*d* XP spectra and **(d)-(f)**, Ni 3*p* spectra for different orientations. Data for all orientations were normalized to the La 4*d* peaks. Open circles show the data points, lines are a guide to the eye obtained by 5-point adjacent average smoothing. Angle-dependent measurements resulting in different mean escape depths *d* qualitatively indicate predominant Ni-termination for the (001) and (111) orientations.

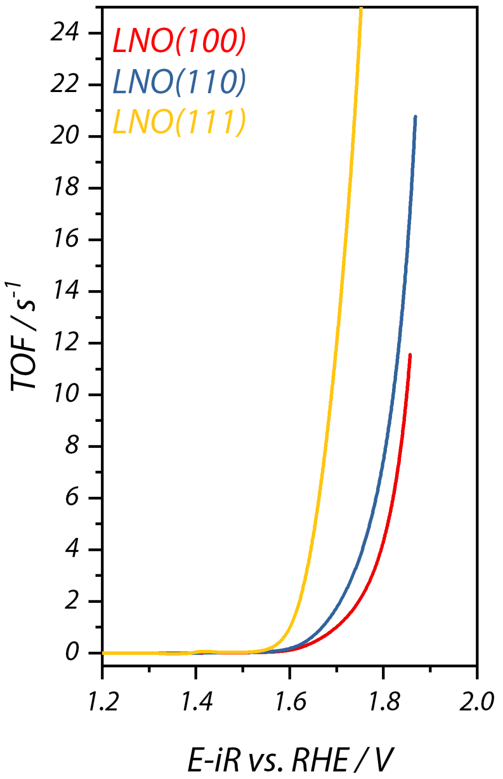

Supplementary Figure 4: Turnover frequency obtained from cyclic voltammetry in the OER potential regime (Fig. 3c in the main text) via normalization of the current density to the number of Ni ions in a perfect perovskite surface.

2 Supplementary Note 2: X-ray absorption spectroscopy

In this section, we discuss the peak positions and related interatomic distances in the EXAFS analysis. The peak positions in Fig. 4c of the main text are lower by about 0.2 to 0.5 Å than the interatomic distances due to a phase shift δ originating from the scattering atoms (note that the EXAFS oscillations are modeled as $\sin(2kR + \delta)$ with wavenumber k) [2].

Peak (i) is assigned to Ni-O with interatomic distance 1.92 Å, while peak (iii) is assigned to Ni-La with interatomic distances of about 3.1 and 3.3 Å and peak (iv) is assigned to Ni-Ni in corner-sharing octahedra with interatomic distance of 3.95 Å, indicative of the perovskite structure [3, 4]. These values are in acceptable agreement with expected values for $LaNiO_{3-\delta}$ (where strain and point defects might increase the lattice parameter compared to the bulk value of 3.84 Å) [5] and with our DFT+U simulation.

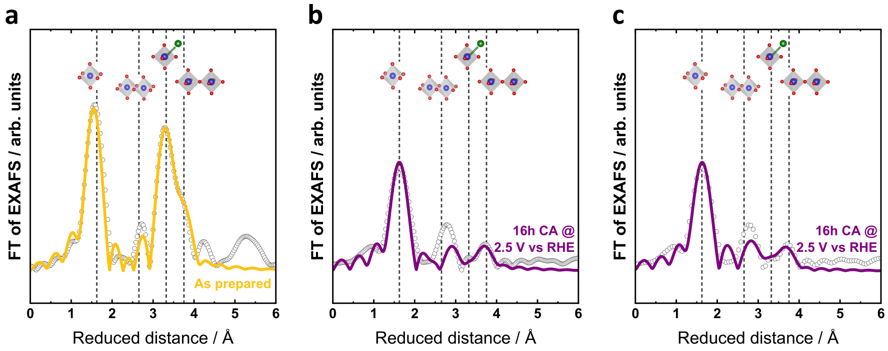

Supplementary Figure 5: Fourier transform of the EXAFS of the Ni-K edge collected on (111)-oriented $LaNiO_{3-\delta}$ samples. **(a)** As prepared. There is spectral intensity near peak (ii). But it is almost fully explained by sidelobes of the Ni-La interactions (peak (iii)). Other contributions could be Ni-O interactions that were not modeled. Fit in Supplementary Table 1. **(b, c)** 16 h CA at 2.5 V vs. RHE. In (b), we removed the Ni-Ni interaction at 3.2 Å, which results in a worse fit than the one in Fig. 4c of the main text. No new parameter optimization was performed after removal of the Ni-Ni interaction from the fit. In (c), we performed parameter optimization (i.e., a new fit), still excluding the Ni-Ni interaction, fit results are shown in Supplementary Table 5. This fit is also worse than the one in Fig. 4c of the main text. These considerations indicate that the spectral intensity near 2.5 Å reduced distance are mainly due to sidelobes for the as prepared sample but due to a (hydr)oxide phase for the sample after 16 h CA at 2.5 V. Data are shown as circles and EXAFS fits as lines. Fourier transforms were performed between 35 and 550 eV (above $E_0 = 8333$ eV) with a cosine window covering the first and last 10% of the data range.

The EXAFS of $LaNiO_{3-\delta}$ after 16 h at 2.5 V is markedly different. The Ni-O

peak (i) is found at larger distance, corresponding to an interatomic distance of 2.01 Å. This increase in Ni-O bond length is indicative of a reduction of Ni. We note that the actual interatomic Ni-O distance in oxyhydroxides ($Ni^{(3+\delta)+}$) is shorter (1.88 Å) [6] than for the perovskite $LaNiO_{3-\delta}$ (1.92 ± 0.01 Å in Supplementary Table 1 to 1.93 Å [3]) so that the larger distance after 16 h at 2.5 V vs RHE supports a reduced hydroxide or oxide, e.g., $Ni(OH)_2$ or NiO. This is expected in our ex-situ measurement, where the samples were returned to open circuit voltage (OCV) and exposed to air before X-ray absorption analysis. According to the Ni-O-H Pourbaix diagram, this would lead to $Ni(OH)_2$ or NiO surface phases after returning to OCV. The interatomic Ni-O distance of 2.01 ± 0.01 Å is slightly shorter than expected for NiO (2.05 - 2.08 Å [6]), suggesting slight oxidation and possibly protonation as in $Ni(OH)_{2-x}$.

Note that each EXAFS peak has sidelobes, and intensity near the distances marked as peak (ii) also results from the sidelopes of peaks (i) and (iii) (Supplementary Fig. 5).[7] For the sample after 16 h CA, there are no major peaks at similar distance, so that a sidelobe can be excluded. The metal-metal distance at peak (ii) is similar to the edge-sharing reference $LiNiO_2$ (Fig. 4b of the main text). Thus, we assigned the new peak/distance to edge-sharing Ni octahedra at 2.98 Å in $LaNiO_{3-\delta}$ after 16 h (Supplementary Fig. 5b,c) and note that the corner-sharing Ni octahedra, i.e., peak (iv), are resolved simultaneously.

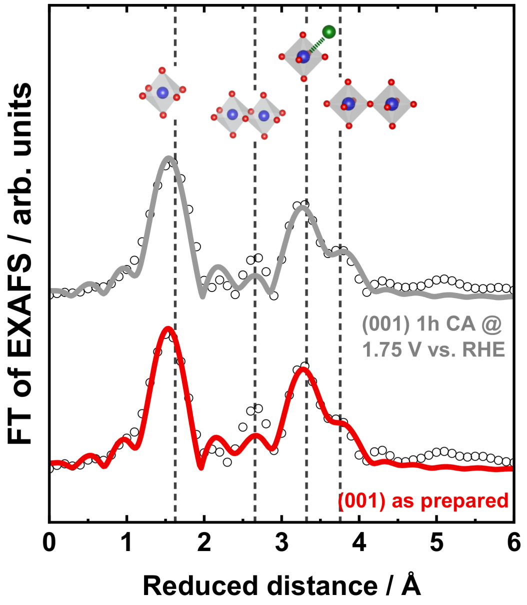

Supplementary Figure 6: Fourier transform of the EXAFS of the Ni-K edge collected on (001)-oriented $LaNiO_{3-\delta}$ samples. Data are shown as circles and EXAFS fits as lines. Fourier transforms were performed between 35 and 550 eV (above $E_0 = 8333\,eV$) with a cosine window covering the first and last 10 % of the data range. Fits in Supplementary Tables 6, 7). The region around 2.5 Å is not well fit, which may suggest that (hydr)oxide is present on the as prepared sample and after 1 h CA at 1.75 V vs RHE.

3 Supplementary Note 3: Additional structural and compositional analysis after OER

To investigate structural and compositional changes as a function of operation time, we performed XRD, XPS and STEM analysis after up to 100 h operation time.

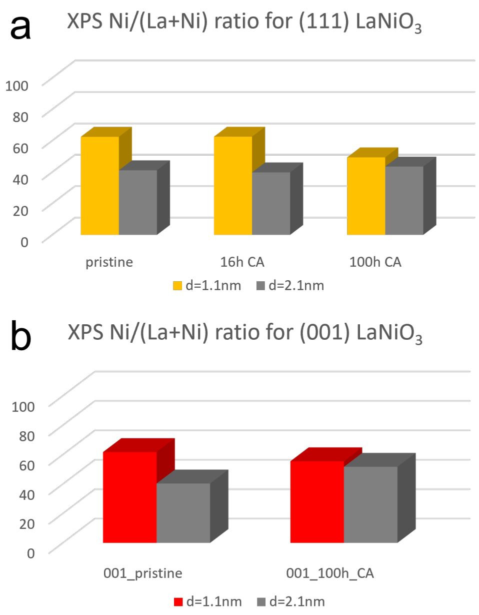

Supplementary Figure 7: Cation ratio for $LaNiO_{3-\delta}$ **(a)** (111) and **(b)** (001) films before and after chronoamperometry (CA) for mean escape depths of $d = 1.1$ nm and $d = 2.1$ nm. Only minor changes in cation composition of the surface layer is observed in the first 16 h of operation.

XPS: We compare the surface and subsurface composition, obtained from angle-dependent XPS measurements (resulting in different mean information depths $d = 1.1$ nm and $d = 2.1$ nm, Supplementary Fig. 6). The overall Ni/La ratio at $d = 1.1$ nm is higher than for $d = 2.1$ nm for all samples, indicating predominant Ni termination in the as-prepared state for both (100) and (111) orientations. For operation up to 16 h, the Ni/La ratio remains constant, as shown exemplarily for the (111) orientation 7a. This indicates that on average, the transformed surface is as Ni-rich as the predominantly Ni-terminated as-prepared state. Only after additional stability testing up to a total operation time of 100 h, we find that for both (100) and (111) samples, the Ni/La ratio changes: While the surface remains slightly Ni-rich, the overall cation ratio approaches 1:1. But we note that the XPS signal intensity decreased by a factor

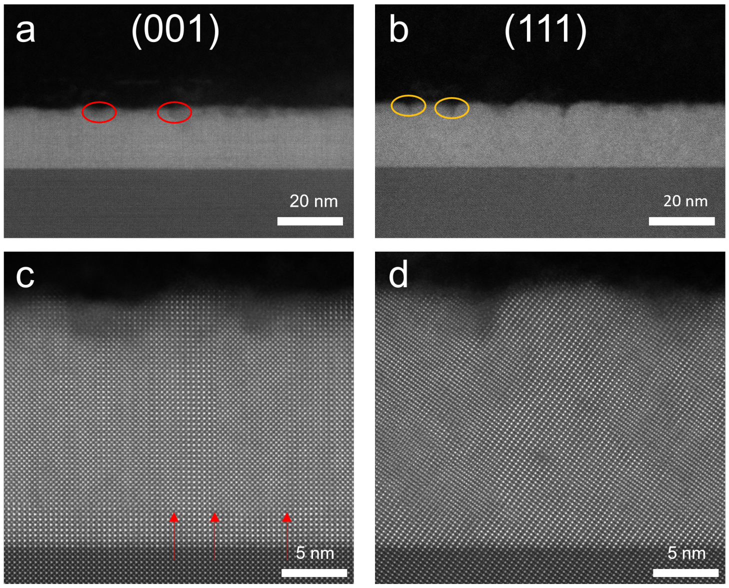

Supplementary Figure 8: HAADF STEM micrographs of $LaNiO_{3-\delta}$ films after 100 h CA for (001) orientation (a, c) and (111) orientation (b,d). Pit-like corrosion is observed after this long-time stability tests (highlighted with red and yellow ellipses in panels a and b). In addition, we still observe the extended defects in the bulk of the film, similar to the samples before CA (examples highlighted with red arrows).

of roughly 10 from the pristine state to the 100 h CA state, indicative of an increased surface roughening (in line with the XRR and TEM results discussed below), making Ni/La ratios extracted from the area-integrated technique after 100 h CA less reliable.

STEM: We performed high-angle annular dark-field imaging (HAADF) STEM analysis of (001) and (111) $LaNiO_{3-\delta}$ samples before and after 100 h CA, to track the most severe changes during stability tests (Supplementary Fig. 7). We note that cross-sectional STEM relies on preparation of lamella using a focussed ion beam (FIB). While we used a protective carbon layer during lamella preparation, the top surface (1-2 unit cells) may be damaged during FIB. Nevertheless, the STEM analysis provides valuable additional information: Even after 100 h of operation, the bulk of the film remained intact (in line with the XRD analysis below). Near the surface, we observe pit-like corrosion: The entire lamellas for both orientations were decorated with nm-sized amorphous regions with a depth of up to 3 nm. These observations are an interesting starting point for future investigations of stability and degradation mechanisms in perovskite-type electrocatalysts, but the observed pits are not directly related to our observations regarding activity.

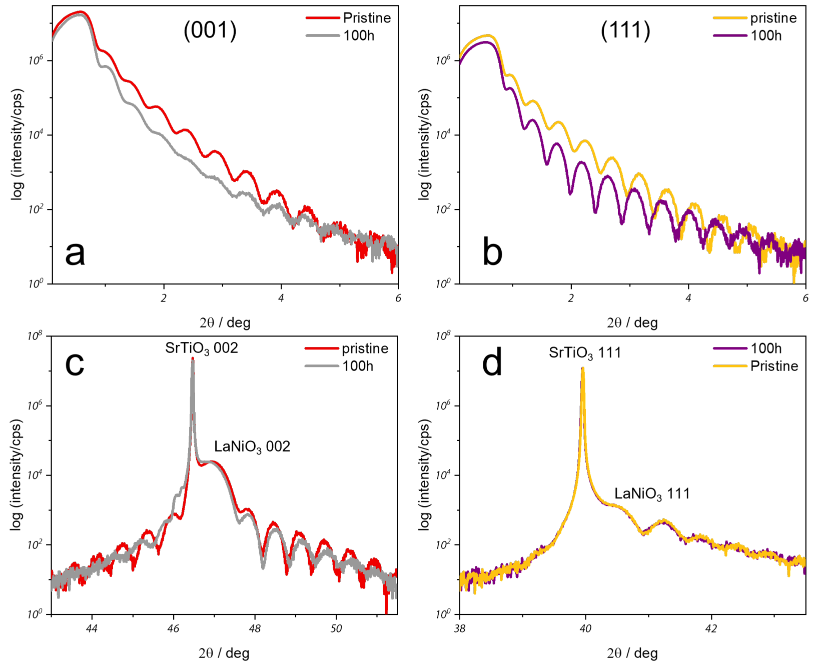

Supplementary Figure 9: XRR and XRD analysis before and after 100 h CA for (001) orientation (a, c) and (111) orientation (b,d).

XRD/XRR: We performed X-ray reflectivity (XRR) and X-ray diffraction (XRD) before and after 100 h CA (Supplementary Fig. 8). Overall, the data confirm that the bulk of the films did not change to a large extent, in line with the STEM analysis. The XRR data is consistent with an increased surface roughening and a similar thickness for all films (with a 1 uc thicker (111) film after 100 h). The nm-sized amorphous regions observed in TEM make the extraction of quantitative figures from XRR difficult. The (bulk-sensitive) XRD data indicates a similarly high crystallinity (similarly pronounced Laue fringes) and similar lattice constant before and after the stability test, with small thickness variations (variations in the period of the Laue fringes).

Summary: We observe local near-surface amorphization after extended stability tests, but otherwise the $LaNiO_{3-\delta}$ samples remain highly-ordered model systems in the bulk. We conclude that the long-time stability observed for the bulk of the films (XRD, STEM) and the comparably stable surface cation ratio (for at least 16 h of operation) form the basis for our detailed analysis of the transformed surface using XAS and DFT+U.

4 Supplementary Note 4: Additional DFT+U results.

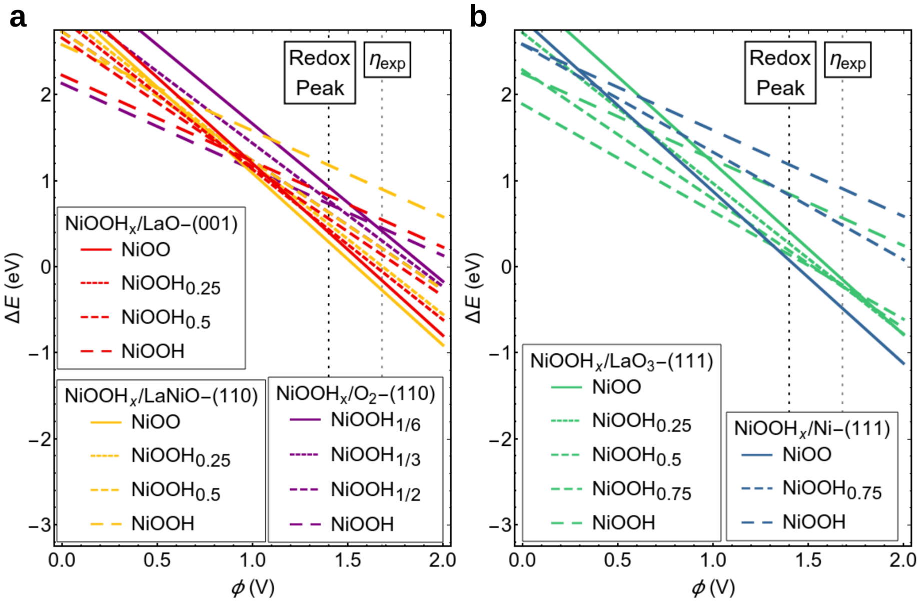

Supplementary Figure 10: Relative stability of $NiOOH_x$ on **(a)** LaO-terminated $LaNiO_3$(001) and LaNiO- and O_2-terminated $LaNiO_3$(110) and **(a)** LaO_3- and Ni-terminated $LaNiO_3$(111) as a function of applied voltage, at pH = 13. x denotes the number of hydrogen atoms intercalated between the transformed NiOO layer and the underlying perovskite. The Hubbard parameter on the Ni $3d$ states is $U = 5.5\,\mathrm{eV}$ in all cases.

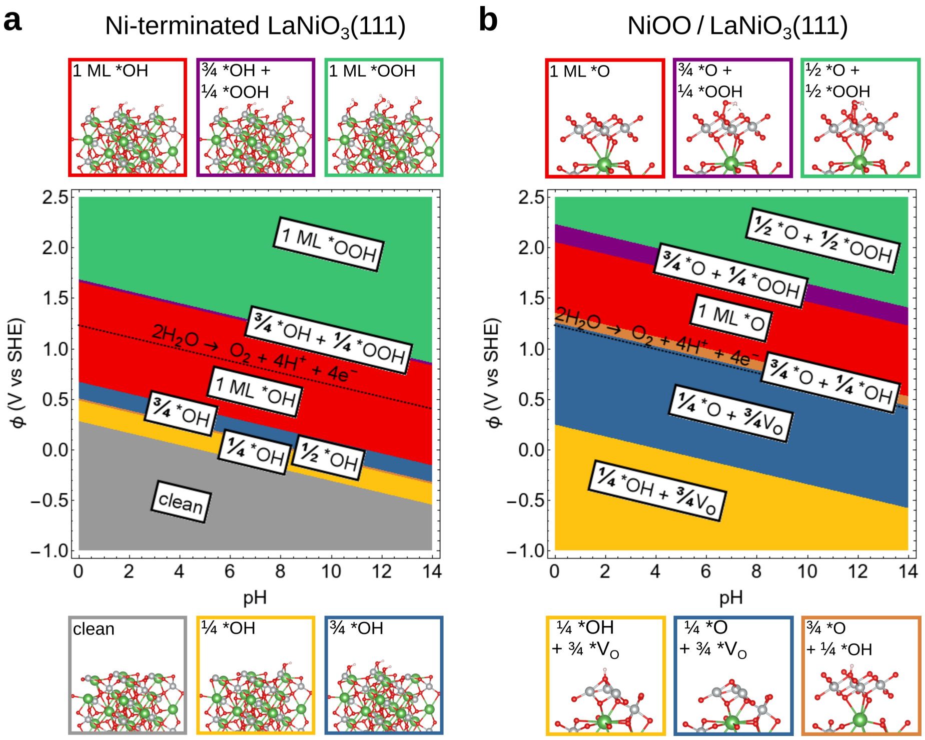

Supplementary Figure 11: Surface Pourbaix diagrams showing the most stable coverages with functional groups as a function of applied voltage and pH for **(a)** untransformed, Ni-terminated $LaNiO_3(111)$. **(b)** a NiOO-layer on LaO_3-terminated $LaNiO_3(111)$. Note that the voltage in experiment is measured vs RHE, therefore the reaction conditions correspond to $\phi = (1.68 - 0.0591 \cdot \text{pH}) = 0.91\,\text{V}$ vs SHE and pH $= 13$ in the diagram. Above and below, side views of the coverages are shown, with frame colors matching the respective areas in the diagram. Note that the calculations for the untransformed and transformed surface are performed with $U = 2.9\,\text{eV}$ and $U = 5.5\,\text{eV}$, respectively (see Supplementary Discussion). Using $U = 2.9\,\text{eV}$ for the latter leads to the same surface termination under OER conditions despite some changes in regions of stability of the terminations.

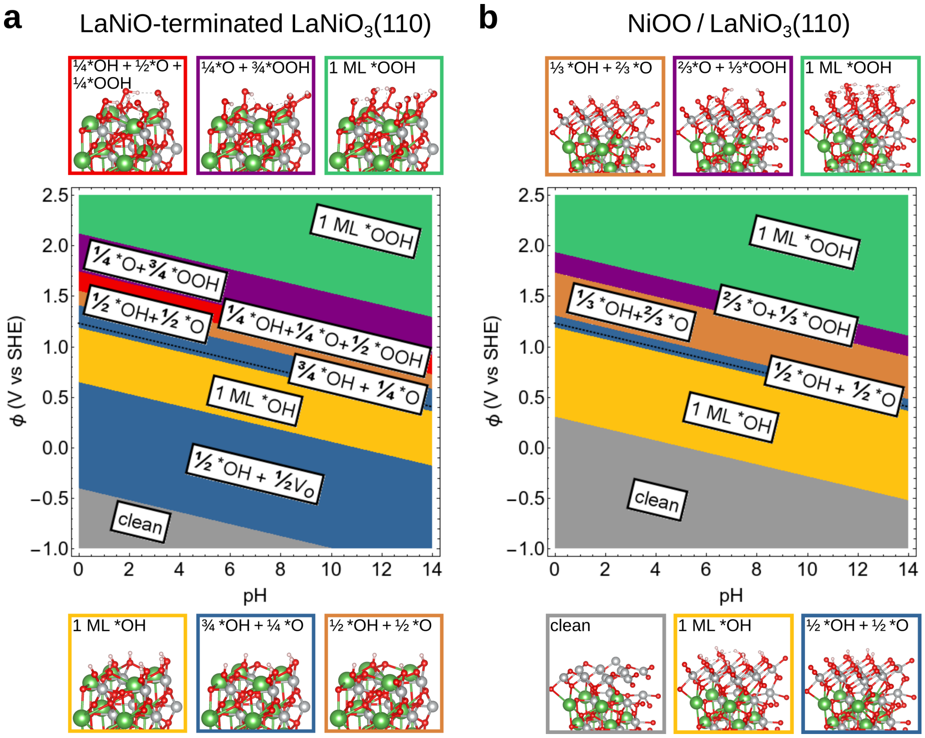

Supplementary Figure 12: Surface Pourbaix diagrams showing the most stable coverages with functional groups as a function of applied voltage and pH for **(a)** untransformed, LaNiO-terminated $LaNiO_3(110)$ (the O_2-termination is treated as a possible coverage). **(b)** a NiOO-layer on LaNiO-terminated $LaNiO_3(110)$. Note that the voltage in experiment is measured vs RHE, therefore the reaction conditions correspond to $\phi = (1.72 - 0.0591 \cdot \mathrm{pH}) = 0.95\,\mathrm{V}$ vs SHE and pH $= 13$ in the diagram. Above and below, side views of the coverages are shown, with frame colors matching the respective areas in the diagram.
Note that the calculations for the untransformed and transformed surface are performed with Hubbard $U = 2.0\,\mathrm{eV}$ and $U = 5.5\,\mathrm{eV}$, respectively (see Supplementary Discussion).

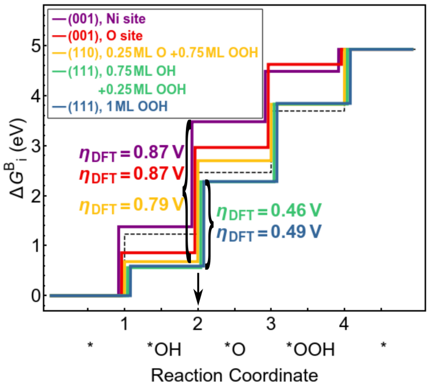

Supplementary Figure 13: Cumulative reaction free energies of NiO_2-terminated (001), LaNiO-terminated (110) and Ni-terminated (111) untransformed perovskite surfaces for Hubbard parameter $U = 2.0\,\mathrm{eV}$. At the (001)-surface all Ni sites are covered by *OH, while a quarter of the O sites are covered by H, as shown in [1]. For (110), the reaction intermediates are adsorbed between one Ni and two La atoms. For (111), the reaction intermediates are adsorbed between one Ni and one La atom. Two surface terminations with functional groups, $0.75\,\mathrm{ML}^*\mathrm{OH} + 0.25\,\mathrm{ML}^*\mathrm{OOH}$ and $1\,\mathrm{ML}\ ^*\mathrm{OOH}$, were taken into account for (111) as both appear close to the experimental overpotential (see Supplementary Fig. 12a). Ideal $\Delta G_\mathrm{i}^\mathrm{B}$ are indicated by dashed black lines.

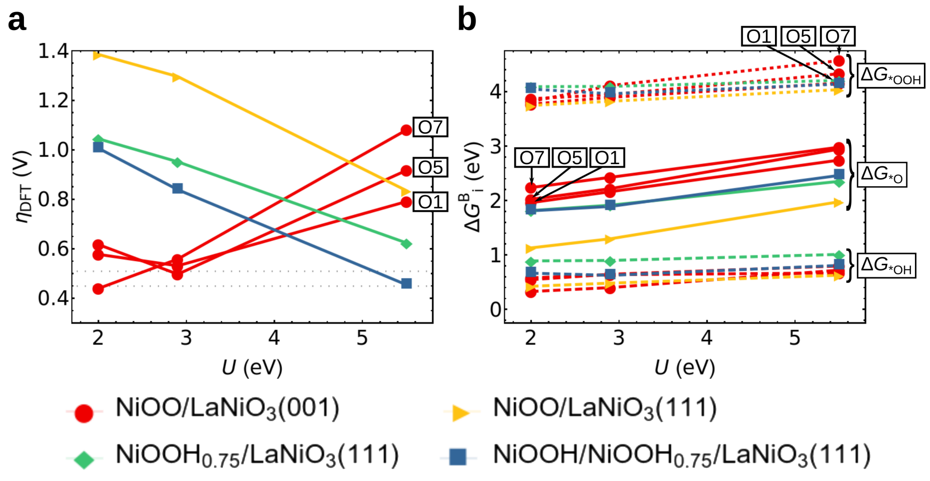

Supplementary Figure 14: **(a)** Dependence of OER overpotential on the Hubbard U parameter. For NiOO/$LaNiO_3$(001), the most favorable reaction sites for each U value are shown (see Supplementary Table 9). Gray, dotted horizontal lines indicate the experimental overpotential of 0.51 V for (001) and 0.45 V for (111) (at 1 mA/cm^2). **(b)** Binding energy of OER intermediates as a function of Hubbard parameter. $\Delta G^{\mathrm{B}}_{\mathrm{O}}$ (solid lines) shows a notably stronger dependence on U than $\Delta G^{\mathrm{B}}_{\mathrm{OOH}}$ (dotted) and $\Delta G^{\mathrm{B}}_{\mathrm{OH}}$ (dashed).

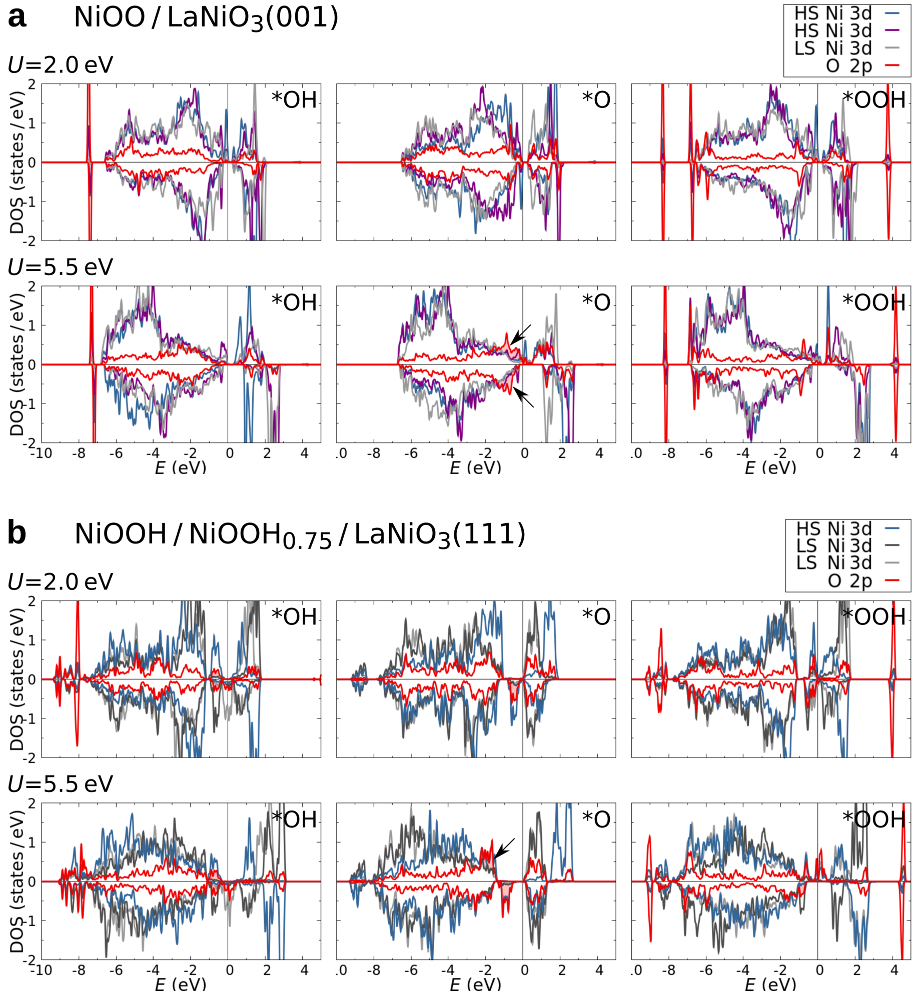

Supplementary Figure 15: Density of states of oxygen $2p$ and the $3d$ orbitals of the three Ni neighbors for the different OER intermediates at **(a)** one oxyhydroxide-like layer on $LaNiO_3(001)$ and **(b)** $NiOOH/NiOOH_{0.75}$ on $LaNiO_3(111)$. At lower U value, there is a stronger hybridization of O and Ni $3d$ states. For $U = 5.5\,\text{eV}$, the occupied Ni $3d$ bands are shifted to lower energies, which reduces the hybridization with the O $2p$ states in the region just below the Fermi edge, especially for the *O intermediate. For the *OH and *OOH intermediates, the effect is much weaker.

5 Supplementary Note 5: Choice of the Hubbard U-parameter

The choice of the Hubbard U-parameter applied to the Ni 3d orbitals turns out to have a considerable effect on the calculated OER overpotentials, as shown for four sample systems in Supplementary Fig. 14a. To gain more insight, we plotted the binding energies of the OER intermediates $\Delta G_{\mathrm{i}}^{\mathrm{B}}$ as a function of U in Supplementary Fig. 14b. On both transformed facets there is only a slight increase of $\Delta G_{*\mathrm{OH}}^{\mathrm{B}}$ and $\Delta G_{*\mathrm{OOH}}^{\mathrm{B}}$ with increasing U, but a markedly stronger one for $\Delta G_{*\mathrm{O}}^{\mathrm{B}}$. This implies that the height of the $^{*}\mathrm{OH} \rightarrow^{*} \mathrm{O}$ step increases, while that of $^{*}\mathrm{O} \rightarrow^{*} \mathrm{OOH}$ decreases with U. While for the transformed (111) surface the PDS remains $^{*}\mathrm{O} \rightarrow^{*} \mathrm{OOH}$ for all U values, for the (001) transformed surface the PDF is $^{*}\mathrm{OH} \rightarrow^{*} \mathrm{O}$ at $U = 5.5\,\mathrm{eV}$, but there is a crossover to $^{*}\mathrm{O} \rightarrow^{*} \mathrm{OOH}$ and a lower overpotential for an increasing number of reaction sites below $U = 2.9$ eV.

To gain further insight into the U dependence of η and why mainly $\Delta G_{*\mathrm{O}}^{\mathrm{B}}$ is strongly influenced by U, we analyzed the projected density of states of oxygen and its Ni neighbors for the three intermediates $\Delta G_{*\mathrm{OH}}^{\mathrm{B}}$, $\Delta G_{*\mathrm{O}}^{\mathrm{B}}$ and $\Delta G_{*\mathrm{OOH}}^{\mathrm{B}}$ for both transformed facets (Supplementary Fig. 15). For *O, we observe a higher density of O 2p states close to the Fermi edge for $U = 2.0\,\mathrm{eV}$. As the Ni states shift to lower energies with increasing U, the hybridization with the O 2p states is reduced, resulting in a weaker bonding, and thus higher $\Delta G_{*\mathrm{O}}^{\mathrm{B}}$. This effect is stronger for the (001)-orientation. In the case of *OH and *OOH the oxygen 2p states are more uniformly distributed and the hybridization with the Ni 3d states is not or only marginally altered with change of U. In other words, the covalency of the Ni-O bonds changes with the U value, where a more ionic bond leads to weak bonding of the *O intermediate.

6 Supplementary Tables

Supplementary Table 1: EXAFS fit results of (111) $LaNiO_{3-\delta}$ as prepared. Phase functions generated from $LaNiO_3$ were used. An asterisk indicates that the parameter was fixed during the fit between 35 - 550 eV in k-space. The filtered square R-factor [8] (0 - 4.5 Å) of this fit was 5.3. Fit errors were calculated as described in the experimental section. Peak position refers to Fig. 4.

#	Ni-O	Ni-La	Ni-La	Ni-Ni	Ni-O
R [Å]	1.92 ± 0.01	3.13 ± 0.02	3.29 ± 0.01	3.95 ± 0.01	4.15 ± 0.04
N	6.1 ± 0.5	1.8 ± 0.9	4.4 ± 1.0	9.9 ± 1.8	22.4 ± 7.3
$2\sigma^2$ [Å^2]	0.005*	0.005*	0.005*	0.005*	0.005*
Peak position [Å]	1.5	3.3	3.3	3.7	3.7

Supplementary Table 2: EXAFS fit results of (111) $LaNiO_{3-\delta}$ after 1 h CA. Phase functions generated from $LaNiO_3$ were used. An asterisk indicates that the parameter was fixed during the fit between 35 - 550 eV in k-space. The filtered square R-factor [8] (0 - 4.5 Å) of this fit was 6.0. Fit errors were calculated as described in the experimental section. Peak position refers to Fig. 4.

#	Ni-O	Ni-La	Ni-La	Ni-Ni	Ni-O
R [Å]	1.92 ± 0.01	3.11 ± 0.02	3.29 ± 0.01	3.95 ± 0.01	4.15 ± 0.04
N	6.0 ± 0.5	1.8 ± 0.8	4.1 ± 1.0	8.2 ± 1.8	20.1 ± 7.2
$2\sigma^2$ [Å^2]	0.005*	0.005*	0.005*	0.005*	0.005*
Peak position [Å]	1.5	3.3	3.3	3.7	3.7

Supplementary Table 3: EXAFS fit results of (111) $LaNiO_{3-\delta}$ after 16 h CA. Phase functions generated from $LaNiO_3$ were used. An asterisk indicates that the parameter was fixed during the fit between 35 - 550 eV in k-space. The filtered square R-factor [8] (0 - 4.5 Å) of this fit was 13.3. Fit errors were calculated as described in the experimental section. Peak position refers to Fig. 4.

#	Ni-O	Ni-La	Ni-La	Ni-Ni
R [Å]	2.010 ± 0.01	3.06 ± 0.02	3.22 ± 0.04	3.96 ± 0.03
N	4.8 ± 0.6	2.0 ± 0.7	1.5 ± 0.8	1.6 ± 0.8
$2\sigma^2$ [Å^2]	0.005*	0.005*	0.005*	0.005*
Peak position [Å]	1.6	2.7	2.7	3.7

Supplementary Table 4: EXAFS fit results of (111) $LaNiO_{3-\delta}$ after 16 h CA at 2.5 V. Phase functions generated from $LaNiO_3$ and NiO were used. An asterisk indicates that the parameter was fixed during the fit between 35 - 550 eV in k-space. The filtered square R-factor [8] (0 - 4.5 Å) of this fit was 13.2. Fit errors were calculated as described in the experimental section. Peak position refers to Fig. 4.

#	Ni-O	Ni-La	Ni-Ni	Ni-Ni
R [Å]	2.01 ± 0.01	3.06 ± 0.02	3.22 ± 0.03	3.96 ± 0.02
N	4.7 ± 0.6	0.6 ± 0.6	1.0 ± 0.6	1.3 ± 0.8
$2\sigma^2$ [Å^2]	0.005*	0.005*	0.005*	0.005*
Phase function	NiO	$LaNiO_3$	NiO	$LaNiO_3$
Peak position [Å]	1.6	2.7	2.7	3.7

Supplementary Table 5: EXAFS fit results of (111) $LaNiO_{3-\delta}$ after 16 h CA at 2.5 V, without hydroxide contribution. Phase functions generated from $LaNiO_3$ were used. An asterisk indicates that the parameter was fixed during the fit between 35 - 550 eV in k-space. The filtered square R-factor [8] (0 - 4.5 Å) of this fit was 15.6. Fit errors were calculated as described in the experimental section. Peak position refers to Fig. 4.

#	Ni-O	Ni-La	Ni-Ni	Ni-Ni
R [Å]	2.013 ± 0.08	3.06 ± 0.02	n/a	3.97 ± 0.03
N	4.8 ± 0.5	0.9 ± 0.3	n/a	1.4 ± 0.8
$2\sigma^2$ [Å^2]	0.005*	0.005*	n/a	0.005*
Peak position [Å]	1.6	2.7	n/a	3.7

Supplementary Table 6: EXAFS fit results of (001) $LaNiO_{3-\delta}$ as prepared. Phase functions generated from $LaNiO_3$ were used. An asterisk indicates that the parameter was fixed during the fit between 35 - 550 eV in k-space. The filtered square R-factor [8] (0 - 4.5 Å) of this fit was 7.3. Fit errors were calculated as described in the experimental section. Peak position refers to Fig. 4.

#	Ni-O	Ni-La	Ni-La	Ni-Ni	Ni-O
R [Å]	1.91 ± 0.09	2.62 ± 0.05	3.29 ± 0.01	3.96 ± 0.01	4.13 ± 0.03
N	5.2 ± 0.6	0.4 ± 0.3	2.2 ± 0.7	7 ± 2	19 ± 9
$2\sigma^2$ [Å^2]	0.005*	0.005*	0.005*	0.005*	0.005*
Peak position [Å]	1.6	3.3	3.3	3.7	3.7

Supplementary Table 7: EXAFS fit results of (001) $LaNiO_{3-\delta}$ after 1 h CA at 1.75 V. Phase functions generated from $LaNiO_3$ were used. An asterisk indicates that the parameter was fixed during the fit between 35 - 550 eV in k-space. The filtered square R-factor [8] (0 - 4.5 Å) of this fit was 7.2. Fit errors were calculated as described in the experimental section. Peak position refers to Fig. 4.

#	Ni-O	Ni-La	Ni-La	Ni-Ni	Ni-O
R [Å]	1.92 ± 0.01	2.71 ± 0.08	3.27 ± 0.02	3.97 ± 0.02	4.15 ± 0.04
N	5.3 ± 0.7	0.3 ± 0.4	2.0 ± 0.8	7 ± 3	20 ± 11
$2\sigma^2$ [Å^2]	0.005*	0.005*	0.005*	0.005*	0.005*
Peak position [Å]	1.6	3.3	3.3	3.7	3.7

Supplementary Table 8: Binding energies of the OER intermediates and overpotential on untransformed $LaNiO_3$ surfaces. All Ni sites at the (001) surface are covered by $^*OH$, while a quarter of the O sites are covered by $^*H$, as shown in [1]. For (110), the functional groups covering the surface are adsorbed between one Ni and two La ions. For (111), functional groups are adsorbed between one Ni and one La ions.

Termination	Coverage	Site	ΔG^{B}_{*OH} (eV)	ΔG^{B}_{*O} (eV)	ΔG^{B}_{*OOH} (eV)	η (V)
NiO_2-$LaNiO_3$(001)	1 ML $^*OH$ + 1/4 ML $^*H$	Ni	1.38	3.48	3.70	0.87
		O	0.86	2.96	4.70	0.87
LaNiO-$LaNiO_3$(110)	3/4 ML $^*OOH$	2 La+Ni	0.68	2.70	3.82	0.79
Ni-$LaNiO_3$(111)	1 ML $^*OOH$	La+Ni	0.59	2.28	3.84	0.46
	3/4 ML $^*OH$ + 1/4 ML $^*OOH$		0.56	2.28	3.83	0.49

Supplementary Table 9: Binding energies of OER intermediates and overpotential for different lattice oxygen reaction sites at NiOO/$LaNiO_3$(001) at Hubbard $U = 5.5\,\mathrm{eV}$. Bond lengths to the three Ni neighbors of the reaction site are given, as well as the Ni magnetic moments. Bond lengths are marked in bold, if they are substantially increased due to distortion, magnetic moments of $\sim 1\,\mu_\mathrm{B}$ are marked in bold. The accompanying figure shows the positions of the reaction sites. To emphasize elongated Ni-O bonds, other bonds are shown semi-transparent.

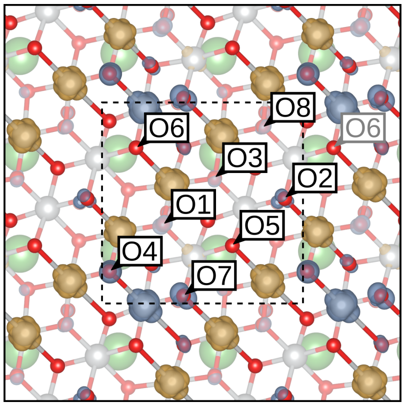

	$d_{\mathrm{Ni-O}}$ (Å), M_{Ni} (μ_B)			$\Delta G^\mathrm{B}_{*\mathrm{OH}}$ (eV)	$\Delta G^\mathrm{B}_{*\mathrm{O}}$ (eV)	$\Delta G^\mathrm{B}_{*\mathrm{OOH}}$ (eV)	η (V)
NiOO/$LaNiO_3$(001)							
O1	1.97, 0.27	1.87, **1.04**	1.90, 0.69	0.73	2.75	4.16	0.79
O2	**2.05**, 0.69	1.88, 0.12	1.88, 0.09	0.68	2.81	4.13	0.90
O3	1.90, 0.09	1.89, **0.97**	1.91, **1.04**	0.72	2.86	4.10	0.91
O4	**2.14**, **-1.00**	1.84, 0.69	1.84, **1.06**	0.61	2.76	4.00	0.91
O5	**2.16**, **1.06**	1.85, 0.09	1.84, 0.27	0.80	2.95	4.33	0.92
O6	**2.25**, **1.04**	1.84, **-1.04**	1.84, 0.12	0.49	2.72	3.92	1.01
O7	**2.32**, **0.97**	1.85, 0.27	1.83, **-1.04**	0.67	2.99	4.58	1.08
O8	1.98, 0.12	1.91, **1.06**	1.88, **0.97**	0.67	3.00	4.30	1.09

Supplementary Table 10: Binding energies of OER intermediates and overpotential for different lattice oxygen reaction sites for NiOO on LaNiO-terminated $LaNiO_3(110)$ at Hubbard $U = 5.5\,\mathrm{eV}$. The transformed surface is covered by 1/3 ML H under reaction conditions, the sites with hydrogen are marked in the table. Bond lengths to the three Ni neighbors of the reaction site are given, as well as Ni magnetic moments. Bond lengths are marked in bold, if they are substantially increased due to distortion, magnetic moments of $\sim 1\,\mu_B$ are marked in bold. The accompanying figure shows the positions of the reaction sites. To emphasize elongated Ni-O bonds, other bonds are shown semi-transparent.

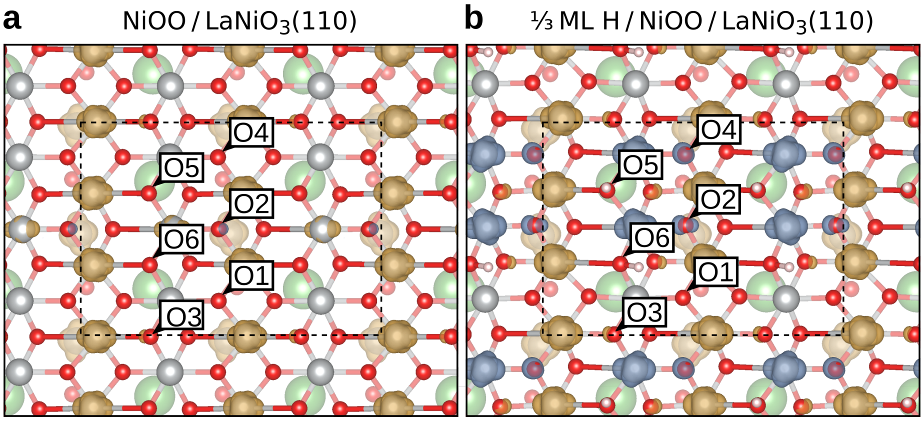

Site	$d_{\mathrm{Ni-O}}$ (Å), M_{Ni} (μ_B)			ΔG^{B}_{*OH} (eV)	ΔG^{B}_{*O} (eV)	ΔG^{B}_{*OOH} (eV)	η (V)
$NiOO/LaNiO_3(110)$							
O1	1.85, 0.41	1.86, **1.00**	1.85, **1.00**	0.55	2.53	4.01	0.74
O2	**2.31**, **1.00**	1.80, 0.41	1.82, 0.04	0.46	2.71	3.81	1.02
O3	**2.17**, **0.96**	1.81, 0.04	1.80, 0.07	0.26	2.35	3.68	0.86
O4	1.96, 0.04	1.86, **1.00**	1.84, **0.96**	0.38	2.19	3.86	0.58
O5	1.96, 0.07	1.84, **0.96**	1.86, **1.00**	0.42	2.55	3.86	0.91
O6	**2.33**, **1.00**	1.83, 0.07	1.80, 0.41	0.51	2.69	3.94	0.95
1/3 ML $H/NiOO/LaNiO_3(110)$							
O1	**1.96**, **-0.90**	1.85, **1.03**	1.83, **1.03**	0.37	1.94	3.65	0.48
O2-H	**2.49**, **1.00**	1.81, **-0.90**	1.87, 0.06	0.63	2.42	3.98	0.56
O3	**2.14**, **1.00**	1.82, 0.14	1.87, **-0.91**	0.42	1.88	3.75	0.64
O4	1.93, 0.14	1.86, **1.03**	1.87, **1.00**	0.38	1.86	3.78	0.69
O5	**2.04**, **-0.91**	1.81, **1.00**	1.83, **1.03**	0.43	1.86	3.89	0.80
O6-H	**2.09**, **0.90**	1.84, **-0.95**	1.83, **-0.91**	0.27	2.32	3.69	0.82

Supplementary Table 11: Binding energies of OER intermediates and overpotential for different lattice oxygen reaction sites on LaO_3-terminated $NiOO/LaNiO_3(111)$, $NiOOH_{0.75}/LaNiO_3(111)$ and $NiOOH/NiOOH_{0.75}/LaNiO_3(111)$ at Hubbard $U = 5.5\,\text{eV}$. Bond lengths to the three Ni neighbors of the reaction site are given, as well as the Ni magnetic moments. Bond lengths are marked in bold, if they are increased due to the Jahn-Teller effect, magnetic moments of $\sim 1\,\mu_B$ are marked in bold. The accompanying figure shows the positions of the reaction sites.

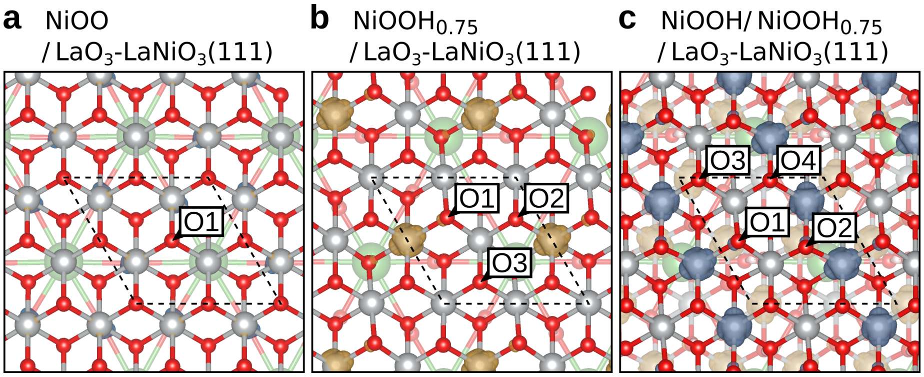

Site	$d_{\text{Ni-O}}$ (Å), M_{Ni} (μ_B)			ΔG^B_{*OH} (eV)	ΔG^B_{*O} (eV)	ΔG^B_{*OOH} (eV)	η (V)
$NiOO/LaNiO_3(111)$							
O1	1.86, 0.21	1.86, 0.21	1.85, 0.08	0.63	1.98	4.04	0.83
$NiOOH_{0.75}/LaNiO_3(111)$							
O1	**2.00**, **0.92**	1.84, -0.02	1.84, -0.02	1.01	2.36	4.21	0.62
O2	1.84, -0.02	1.86, **0.92**	1.85, 0.03	0.72	2.12	4.14	0.79
O3	1.86, 0.03	1.86, -0.02	1.86, -0.02	0.91	2.13	4.18	0.82
$NiOOH/NiOOH_{0.75}/LaNiO_3(111)$							
O1	**2.01**, **-0.96**	1.83, 0.01	1.82, 0.01	0.81	2.46	4.14	0.45
O2	1.80, 0.01	1.83, **-0.96**	**1.98**, **-0.95**	0.56	2.38	4.03	0.60
O3	1.87, **-0.95**	1.87, 0.01	1.85, 0.01	0.55	2.07	4.00	0.70
O4	1.85, 0.01	1.86, **-0.95**	1.84, **-0.96**	0.73	2.26	4.10	0.61

Supplementary Table 12: Binding energies of OER intermediates and overpotential for different lattice oxygen reaction sites on Ni-terminated NiOO/Ni-$LaNiO_3$(111), and NiOOH/NiOO/Ni-$LaNiO_3$(111) at Hubbard $U = 5.5\,\mathrm{eV}$. Bond lengths to the three Ni neighbors of the reaction site are given, as well as the Ni magnetic moments. Bond lengths are marked in bold, if they are increased due to the Jahn-Teller effect, magnetic moments of $\sim 1\,\mu_\mathrm{B}$ are marked in bold. The accompanying figure shows the positions of the reaction sites.

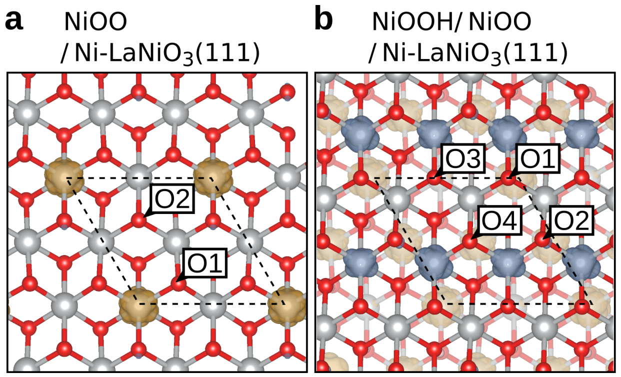

Site	$d_{\mathrm{Ni-O}}$ (Å), M_{Ni} (μ_B)			$\Delta G^{\mathrm{B}}_{*\mathrm{OH}}$ (eV)	$\Delta G^{\mathrm{B}}_{*\mathrm{O}}$ (eV)	$\Delta G^{\mathrm{B}}_{*\mathrm{OOH}}$ (eV)	η (V)
NiOO/Ni-$LaNiO_3$(111)							
O1	1.91, **1.06**	1.85, 0.10	1.83, 0.06	0.18	1.71	3.60	0.66
O2	1.86, 0.09	1.86, 0.06	1.87, 0.10	1.10	2.32	4.48	0.92
NiOOH/NiOO/Ni-$LaNiO_3$(111)							
O1	1.86, 0.00	1.88, **-0.97**	1.84, 0.00	0.76	2.49	4.11	0.50
O2	1.85, **-0.95**	1.81, 0.00	**1.98, -.97**	0.71	2.45	4.12	0.52
O3	1.86, 0.00	1.86, **-0.95**	1.85, 0.00	0.77	2.29	4.07	0.56
O4	1.84, **-0.97**	1.85, 0.00	1.90, **-0.95**	0.72	2.17	4.10	0.71

Supplementary Table 13: Total number N of Ni ions in oxidation states 2+, 3+ and 4+ for the different OER intermediates at the transformed surfaces. Note that Ni^{2+}, with a corresponding magnetic moment of $\sim 1.5\,\mu_\mathrm{B}$, emerges only at the transformed (110) facet.

Intermediate	(001)		(110)			(110), 1/3 ML H			Ni-(111)		LaO_3-(111)	
	N_{3+}	N_{4+}	N_{2+}	N_{3+}	N_{4+}	N_{2+}	N_{3+}	N_{4+}	N_{3+}	N_{4+}	N_{3+}	N_{4+}
*	5	3	1	8	3	2	8	2	4	0	4	0
*OH	4	4	0	7	5	1	9	2	3	1	4	0
*O	4	4	0	6	6	0	10	2	2	2	2	2
*OOH	4	4	0	7	5	1	9	2	3	1	4	0

Supplementary Table 14: Total vibrational contributions $\Delta(\Delta G - \Delta E)$ to the adsorption energies, consisting of change in zero-point energy ZPE, stored heat $C_V \cdot T$ and entropic term $T \cdot \Delta S$ (both at room temperature $T = 298$ K). Hubbard $U = 2.0$ eV was used on Ni 3d. Literature values are given for comparison.

Adsorbate	Zero-Point Energy ZPE (eV)		Heat $C_V \cdot T$ (eV)		Entropy $T \cdot \Delta S$ (eV)		Total Correction $\Delta(\Delta G - \Delta E)$ (eV)	
*OH	0.414	0.389 [1]	0.027	0.032 [1]	0.047	0.047 [1]	0.3698	0.3613 [1]
*O	0.083	0.098 [1]	0.021	0.017 [1]	0.026	0.023 [1]	0.0324	0.0545 [1]
*OOH	0.480	0.478 [1]	0.076	0.066 [1]	0.152	0.112 [1]	0.3346	0.3818 [1]

Supplementary Table 15: Individual contributions of molecules to total vibrational correction at $T = 298$ K and $p = 0.035$ bar for H_2O and $p = 1$ bar for H_2. Literature values are given for comparison.

Molecule	Zero-Point Energy ZPE (eV)		Heat $C_V \cdot T$ (eV)		Entropy $T \cdot \Delta S$ (eV)	
H_2O	0.569	0.560 [1]	0.103	0.103 [1]	0.670	0.675 [1]
H_2	0.269	0.268 [1]	0.090	0.0905 [1]	0.402	0.408 [1]

Supplementary Table 16: Individual contributions and total vibrational correction for *OH and *O between $LaNiO_3(111)$ and NiO(O) at $T = 298$ K. Hubbard $U = 5.5$ eV was used at the Ni 3d sites.

Adsorbate	ZPE (eV)	$C_V \cdot T$ (eV)	$T \cdot \Delta S$ (eV)	$\Delta(\Delta G - \Delta E)$ (eV)
*OH	0.502	-0.025	-0.083	0.5366
*O	0.108	0.012	0.012	0.0643

References

[1] Baeumer, C. *et al.* Tuning electrochemically driven surface transformation in atomically flat $LaNiO_3$ thin films for enhanced water electrolysis. *Nat. Mater.* **20**, 674–682 (2021).

[2] Newville, M. Fundamentals of XAFS. *Rev. Mineral. Geochem.* **78**, 33–74 (2014).

[3] Ravel, B. Muffin-tin potentials in EXAFS analysis. *J. Synchrotron Radiat* **22**, 1258–1262 (2015).

[4] Levitz, P., Crespin, M. & Gatineau, L. Reduced forms of $LaNiO_3$ perovskite. Part 2.—X-ray structure of $LaNiO_2$ and extended X-ray absorption fine structure study: local environment of monovalent nickel. *J. Chem. Soc., Faraday Trans. 2* **79**, 1195–1203 (1983).

[5] Wold, A., Post, B. & Banks, E. Rare Earth Nickel Oxides. *J. Am. Chem. Soc.* **79**, 4911–4913 (1957).

[6] Risch, M. *et al.* Nickel-oxido structure of a water-oxidizing catalyst film. *Chem. Commun.* **47**, 11912–11914 (2011).

[7] Risch, M. *et al.* Structural changes of cobalt-based perovskites upon water oxidation investigated by EXAFS. *J. Phys. Chem. C* **117**, 8628–8635 (2013).

[8] Calvin, S. *XAFS for Everyone (1st ed.)* (CRC Press, 2013).
